# Chloroplast two-component systems: evolution of the link between photosynthesis and gene expression

**DOI:** 10.1098/rspb.2008.1426

**Published:** 2009-02-25

**Authors:** Sujith Puthiyaveetil, John F. Allen

**Affiliations:** School of Biological and Chemical Sciences, Queen Mary, University of LondonMile End Road, London E1 4NS, UK

**Keywords:** cytoplasmic inheritance, endosymbiosis, redox response regulator, redox sensor kinase, signal transduction, transcription

## Abstract

Two-component signal transduction, consisting of sensor kinases and response regulators, is the predominant signalling mechanism in bacteria. This signalling system originated in prokaryotes and has spread throughout the eukaryotic domain of life through endosymbiotic, lateral gene transfer from the bacterial ancestors and early evolutionary precursors of eukaryotic, cytoplasmic, bioenergetic organelles—chloroplasts and mitochondria. Until recently, it was thought that two-component systems inherited from an ancestral cyanobacterial symbiont are no longer present in chloroplasts. Recent research now shows that two-component systems have survived in chloroplasts as products of both chloroplast and nuclear genes. Comparative genomic analysis of photosynthetic eukaryotes shows a lineage-specific distribution of chloroplast two-component systems. The components and the systems they comprise have homologues in extant cyanobacterial lineages, indicating their ancient cyanobacterial origin. Sequence and functional characteristics of chloroplast two-component systems point to their fundamental role in linking photosynthesis with gene expression. We propose that two-component systems provide a coupling between photosynthesis and gene expression that serves to retain genes in chloroplasts, thus providing the basis of cytoplasmic, non-Mendelian inheritance of plastid-associated characters. We discuss the role of this coupling in the chronobiology of cells and in the dialogue between nuclear and cytoplasmic genetic systems.

## 1. Two-component systems enter the eukaryotic domain of life

**Figure 1:**
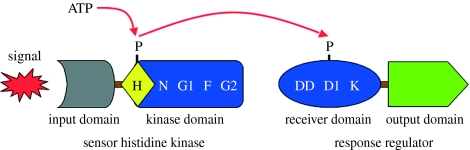
Schematic of a two-component system. The sensor kinase and the response regulator components and their domain architecture are shown. The kinase domain consists of a dimerization domain (yellow diamond) and an ATP-binding domain (blue rectangle). The H, N, G1, F and G2 motifs of the kinase domain are indicated. Conserved sequence motifs of the response regulator receiver domain, DD, D1 and K, are also indicated. The phosphotransfer signalling mechanism of the two-component systems is depicted as phosphate group transfer from ATP to the conserved aspartate residue of the response regulator via the conserved histidine residue of the sensor kinase.

The name ‘two-component system’ is used to describe members of a class of signal transduction pathways found in eubacteria and made up of two conserved protein components (Stock *et al*. 1985; Nixon *et al*. 1986). These two conserved protein components are a sensor kinase and a response regulator (figure 1).

Of these two, the component that is first to detect and respond to an environmental change is the sensor kinase. A sensor kinase is a histidine protein kinase that combines a variable sensor domain with an invariable kinase domain (figure 1). The sensor domain perceives different specific signals in different histidine sensor kinases. The nature of the signal sensed and the structure of the protein's sensor domain are specific for each histidine sensor kinase. By contrast, the kinase domain is highly conserved in structure and function, being made up of an independent dimerization motif and a catalytic core. The catalytic core of the kinase domain consists of five conserved amino acid motifs: H-box, N, G1, F and G2 (figure 1). The H-box contains the conserved histidine residue that is the site of phosphorylation and is usually located in the dimerization motif. N, G1, F and G2 boxes form the ATP-binding pocket of the catalytic core (Stock *et al*. 2000).

The second component of any two-component system is its response regulator. The response regulator protein is also made up of two domains (figure 1). The first domain of a response regulator is its invariable receiver domain. The second domain of a response regulator is its variable effector domain, which mediates the specific output response. The chemistry of signal transduction is common to different pathways: two-component systems use a phosphotransfer mechanism from the invariant kinase domain of the sensor to the invariant receiver domain of the response regulator (figure 1). The sequence of events that leads to an output response from a two-component signalling pathway begins when histidine kinases, in their functional dimeric form, and upon sensing the signal, undergo an ATP-dependent trans-autophosphorylation reaction, whereby one histidine kinase monomer phosphorylates a second monomer within the dimer. The phosphate group becomes covalently, though weakly, bound to a conserved histidine residue of the catalytic core. The receiver domain of the response regulator protein then catalyses the transfer of the phosphate group from the histidine residue of the kinase to a conserved aspartate residue within the receiver domain of the response regulator protein. This creates a high-energy acyl phosphate that activates the effector domain of the response regulator (Stock *et al*. 2000).

There are actually two reactions catalysed by the ‘sensor kinase’ enzyme. The first is transfer of the *γ*-phosphate of ATP to a histidine side chain of the protein itself, to form a phosphoamide linkage (autophosphorylation; equation (1.1)). The second reaction is transfer of the phosphate moiety from the histidine of the sensor kinase to an aspartate on the corresponding response regulator (equation (1.2)). Thus, the phosphohistidine acts as a covalent chemical intermediate in transfer of the phosphate group between ATP and the response regulator, and so sensor kinases are, in a biochemical sense, really ‘response regulator kinases’ (equation (1.3)).

(1.1) Sensor‐His+ATP⇌Sensor‐His∼ADP

(1.2) Regulator‐Asp+Sensor‐His∼P⇌Regulator‐Asp∼P+Sensor‐His

(1.3) Sum:Regulator‐Asp+ATP⇌Regulator‐Asp∼P+ADP

The histidine sensor kinase becomes autophosphorylated (equation (1.1)) if, and only if, the specific environmental precondition is met, and on an invariant histidine residue. The phosphate group is then transferred to the aspartate residue of one or more response regulators (equation (1.2)). Phosphorylation of the response regulator then activates the appropriate response to the environmental change that produced the original phosphorylation on histidine.

Although many two-component systems follow the above archetype in modular design (figure 1) and phosphotransfer mechanism, variations exist on this basic theme. These variations may involve additional conserved domains in histidine kinases or separate modules, and more complex phosphorelay pathways.

Two-component systems are known to regulate diverse physiological responses in bacteria. Most of these physiological responses involve transcriptional regulation, mediated by the response regulator protein acting as a transcription factor. These signalling systems must have originated very early in evolution (Stock *et al*. 1989) and are now found in both eubacteria and archaebacteria (‘Archaea’; Koretke *et al*. 2000). The two-component systems are nevertheless typically found in eubacteria, including alpha-proteobacteria and cyanobacteria, and probably became widespread in the eukaryotic domain of life through symbiotic, lateral gene transfer from the ancestors of chloroplasts and mitochondria (Koretke *et al*. 2000).

Chloroplasts and mitochondria retain functional genomes and each houses a complete, cytoplasmic apparatus of gene expression that is separate from that of the nucleus and cytosol. The two-component systems that couple regulatory signals to gene expression were predicted to be present in these eukaryotic subcellular organelles (Allen 1993*a*). The regulatory signals that thus modulate organellar gene expression are now known, notably as changes in the redox state of key components of energy transduction (Pfannschmidt *et al*. 1999*a*). If chloroplasts and mitochondria were responsible for the acquisition of two-component systems by eukaryotes, it is interesting to ask whether these organelles themselves retain the two-component systems from their bacterial ancestors. The answer for chloroplasts is now known. It is ‘yes’. The two-component systems from cyanobacteria have survived in chloroplasts as products of both chloroplast and nuclear genes (Duplessis *et al*. 2007; Puthiyaveetil & Allen 2008*a*; Puthiyaveetil *et al*. 2008). Here, we describe the properties of two-component systems of chloroplasts, their phylogenetic distribution and the functional implications of these analyses for regulatory coupling between photosynthesis and gene expression.

## 2. Component 1. Chloroplast sensor kinases come in different hues

Sequencing of chloroplast genomes is a routine exercise in molecular systematics of plants and algae. Chloroplast DNA sequencing has thus resolved many cladistic disputes. It has also revealed some unexpected genes in chloroplasts, such as subunits of NAD(P)H dehydrogenase. Other unexpected genes show sequence similarity to genes for regulatory proteins that were once thought to be confined to bacteria. One of these bacterial-type regulatory proteins is a histidine sensor kinase variously known as Dfr in *Gracilaria tenuistipitata*, Tsg1 in *Heterosigma akashiwo* and as ycf26 in *Porphyra purpurea* (Duplessis *et al*. 2007). As more plastid genomes were sequenced, it became apparent that this histidine sensor kinase gene was limited in its phylogenetic distribution: *ycf26* is found only in red algal, raphidophyte and haptophyte chloroplasts, and is completely unknown in chloroplasts of green algae and land plants (table 1; Duplessis *et al*. 2007). The phylogenetic distribution of *ycf26* in non-green algae is also found to be discontinuous, as it is absent from chloroplasts of the ancient red alga *Cyanidioschyzon merolae* and from chloroplasts of the diatoms *Phaeodactylum tricornutum* and *Thalassiosira pseudonana* (table 1; Duplessis *et al*. 2007).

Although chloroplast-encoded sensor kinases are unknown in green algae and in land plants, the sequencing of their nuclear genomes has now revealed many genes encoding two-component proteins. In the model higher plant *Arabidopsis thaliana*, there are 54 such genes and at least 16 of them encode putative histidine kinases. The possibility of one of these histidine kinase gene products being targeted to the chloroplast has been examined (Forsberg *et al*. 2001), but none was thought to possess a chloroplast-targeting sequence (Oelmüller *et al*. 2001; Lopez-Juez & Pyke 2005; Wagner & Pfannschmidt 2006). However, we recently identified a nuclear-encoded sensor kinase in *Arabidopsis* chloroplasts (Puthiyaveetil *et al*. 2008). Other investigators might have missed this chloroplast sensor kinase (CSK), because its gene is annotated ‘unknown protein’ and not even included as one of the 16 putative histidine kinase genes of *Arabidopsis*. The CSK gene, in contrast to the ycf26 sensor of non-green algae, has a wide phylogenetic distribution with recognizable homologues in all lineages of green algae and plants, and also in some red algae and diatoms (table 1; Puthiyaveetil *et al*. 2008).

**Figure 2:**
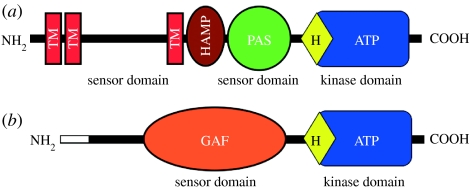
Predicted domain composition of chloroplast sensor kinases, (*a*) ycf26 and (*b*) CSK. Individual domains are labelled as follows: TM, transmembrane; HAMP, domain present in histidine kinases, adenylyl cyclases, methyl-accepting proteins and phosphatases; PAS, domain named after three proteins it occurs in, per, arnt and sim; GAF, domain first described for vertebrate cGMP-specific phosphodiesterase, a cyanobacterial adenylate cyclase and the bacterial formate hydrogen lyase transcription activator FhlA. The white rectangle at the amino terminus of CSK represents the chloroplast transit peptide.

**Figure 3:**
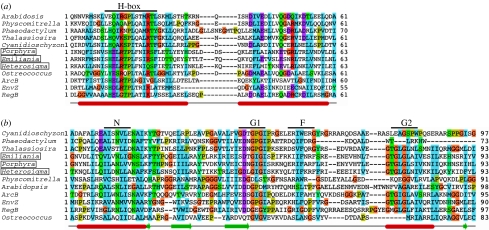
Chloroplast sensor kinases show molecular evolution in their kinase domains. (*a*) Multiple sequence alignment of ycf26 and CSK HisKA domain (dimerization and phosphoacceptor domain as defined by SMART database) from representative species with the three canonical histidine kinases from bacteria, ArcB, EnvZ and RegB. Species names of ycf26 sequences are boxed. Predicted secondary structures are shown at the bottom. Alpha helices are shown as cylinders, beta sheets as thick arrows and the line connecting them as loops and turns. The site of autophosphorylation, H-box, located in the first helix of HisKA domain, is indicated on the top of the alignment. (*b*) The ATP-binding domain is conserved in all chloroplast sensor kinases. ATP-binding domain of representative ycf26, CSK sequences are aligned with those of three canonical histidine kinases, ArcB, RegB and EnvZ. Species names of ycf26 sequences are boxed. N, G1, F and G2 motifs of the ATP-binding domain are shown on top and the predicted secondary structures are shown at the bottom. Alignment was generated with ClustalW (Chenna *et al*. 2003) and edited with Jalview (Clamp *et al*. 2004). Secondary structures are predicted with JNet (Cole *et al*. 2008).

As it stands today, chloroplasts seem to have at least two sensor kinases, one plastid-encoded and the other nuclear-encoded (table 1; Duplessis *et al*. 2007; Puthiyaveetil *et al*. 2008). These two CSKs, although quite different in some sequence features, show some commonalities in their functional design. The chloroplast-encoded sensor kinase of non-greens, ycf26, seems to be a transmembrane sensor as most of its examples have two to three predicted transmembrane helices. The predicted topology (figure 2) of this protein includes one or two transmembrane helices at the N-terminus and a lumenal loop of 120–130 amino acids followed by another transmembrane helix. The predicted stromal-exposed domain of the enzyme has an HAMP linker domain and a PAS domain followed by a kinase domain (figure 2). HAMP domains are known to act as linkers that connect the periplasmic sensor domain with the cytoplasmic or stromal surface-exposed kinase domain of transmembrane histidine kinases (Aravind & Ponting 1999). PAS domains are well known for their role as internal sensors of the redox state (Taylor & Zhulin 1999). The kinase domain of ycf26 is typical of histidine sensor kinases, as it possesses all known sequence motifs for dimerization and transphosphorylation reactions (figure 3). The ycf26 sensor is seen in its minimal form in the raphidophycean alga, *H. akashiwo* (Duplessis *et al*. 2007) and in the cryptophycean alga, *Rhodomonas salina*. In these two species, ycf26 is likely to be a soluble stromal protein with only the PAS sensor domain and the kinase domain. It appears that in most ycf26 proteins the predicted lumenal loop and the internal PAS domain act as two separate sensor domains (Morrison *et al*. 2005).

CSK, in contrast to ycf26, has no predicted membrane-spanning region (figure 2) and seems to be a soluble stromal protein. The sensor domain in CSK is a putative GAF domain, which, as a PAS domain, is implicated in redox sensing (Kumar *et al*. 2007; Vuillet *et al*. 2007). The kinase domain in different CSKs shows variation with respect to the conserved histidine residue in the H-box (figure 3). CSKs from red algae and diatoms have the conserved histidine seen in bacterial sensor kinases, while the green algal CSKs have a tyrosine in place of the histidine residue (figure 3). The moss *Physcomitrella patens* (Rensing *et al*. 2008) and all higher plants have a glutamate residue instead of the conserved histidine in their CSKs (figure 3). While there are precedents for replacement of histidine residues in sensor kinases (Yeh & Lagarias 1998; Moussatche & Klee 2004), this is the first report, to our knowledge, of such a replacement occurring more than once in the evolution of a sensor kinase. This amino acid substitution is reflected in an altered phosphoryl group chemistry in the *Arabidopsis* CSK (Puthiyaveetil *et al*. 2008). Another interesting feature of CSK is that the cytosolically synthesized CSK precursor protein and the mature chloroplast CSK protein have the same molecular mass (Puthiyaveetil *et al*. 2008). This means that the transit peptide is retained in the mature protein, where it may play a role in CSK function.

## 3. Component 2. Chloroplast response regulators: from the known to the unknown

**Figure 4:**
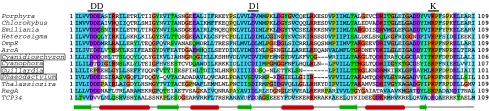
Conserved sequence features of chloroplast response regulators. The receiver domain of the representatives ycf27, ycf29 and the *Arabidopsis* TCP34 are aligned with those of three canonical response regulators in bacteria ArcA, RegA and OmpR. Species names of ycf29 sequences are boxed. Predicted secondary structures are shown at the bottom. Conserved sequence motifs of the receiver domain, DD, D1 and K, are indicated on top of the alignment. The fully conserved D1 motif, which lies in the loop connecting the third beta sheet and the third helix, is the conserved aspartate residue that receives the phosphate group from the phosphohistidine of the sensor kinase. *Arabidopsis* TCP34 shows a moderately conserved receiver domain with the ‘K motif’ still being present, but not entirely aligned with those from other response regulators.

The first report of a chloroplast response regulator, like that of a CSK, came as a simple consequence of systematically sequencing chloroplast genomes. The first chloroplast response regulator was described in red algae and has a high sequence similarity to the bacterial OmpR response regulator transcription factor (Kessler *et al*. 1992). This chloroplast response regulator is generally known as ycf27, as ‘OmpR-like protein’, or simply as ‘OmpR’. It has also been called ‘transcriptional regulatory gene 1’ (trg1) in *H. akashiwo* (Jacobs *et al*. 1999). ycf27 is found in many non-green algal lineages (table 2; Duplessis *et al*. 2007). However, among more than 75 sequenced chloroplast genomes of green algae and land plants, only the charophyte *Chlorokybus atmosphyticus* has the *ycf27* gene (Duplessis *et al*. 2007). The predicted domain architecture of ycf27 conforms to known domain features of response regulators. The receiver domain is typical in having three sequence motifs (figure 4), including the invariant aspartate residue that receives phosphate from the histidine residue of a sensor kinase. The effector domain of ycf27 is a winged helix-turn-helix motif. This motif is a variation of the well-known DNA-binding helix-turn-helix domain. In addition to binding DNA, winged helix-turn-helix motifs also sometimes interact with RNA polymerase to influence gene transcription (Martinez-Hackert & Stock 1997).

A second chloroplast response regulator identified as a by-product of chloroplast genome sequencing is ycf29. This chloroplast response regulator shows high sequence similarity to the NarL response regulator transcription factor of *Escherichia coli* (Maris *et al*. 2002). ycf29 is also known as tctD in *G. tenuistipitata* or as NarL-like transcriptional regulator. ycf29 occurs together with ycf27 in some, but not all, non-green algae (table 2). Like ycf27, ycf29 appears to be limited in its phylogenetic distribution to non-green algae. The ycf29 protein has very similar properties to the ycf27 response regulator (figure 4), with a small difference in their effector domains. ycf29 does not have the extended wing structure in its DNA-binding helix-turn-helix domain.

Most chloroplasts in the green lineages contain no gene for any response regulator. This conclusion does not preclude the existence of response regulators in their chloroplasts as products of nuclear genes, as shown for CSKs (Puthiyaveetil *et al*. 2008). Homologues of ycf27 or ycf29 cannot be readily identified in sequenced nuclear genomes of green algae and plants. Yet it is premature to conclude that nuclear-encoded ycf27 and ycf29 homologues are absent from chloroplasts of green algae and land plants. One possibility is that the chloroplast response regulators in greens are modified so as to accommodate input from a modified histidine kinase such as CSK. The coevolution of sensor kinases and response regulators in a cognate pair is well documented (Koretke *et al*. 2000; Skerker *et al*. 2008). One or more modified response regulators might thus have evaded identification by conventional sequence-similarity searches. Along these lines, a nuclear-encoded, modified response regulator protein, TCP34, has been identified in higher plant chloroplasts (Weber *et al*. 2006). This protein seems to be conserved in all sequenced plant genomes. Our analysis (table 2) identifies a homologue of TCP34 in the moss, *P. patens*. *Physcomitrella* also has a paralogue of TCP34 (Andrew Cuming, University of Leeds, personal communication). We also identify (table 2) single homologues of TCP34 in the nuclear genome of chlorophycean algae, *Ostreococcus tauri* and *Ostreococcus lucimarinus*, but, interestingly, not in *Chlamydomonas reinhardii*. The moss and algal TCP34 homologues are localized in the chloroplast, according to subcellular prediction programmes (results not shown).

The TCP34 protein combines a moderately conserved receiver domain (figure 4) with a tetratricopeptide repeat (TPR) as an effector domain (Weber *et al*. 2006). It also seems that a part of the receiver domain of this protein may be co-opted as a DNA-binding protein with a helix-turn-helix motif. It has also been demonstrated that TCP34 is phosphorylated, as was discovered in the course of a search for a specific chloroplast protein kinase (Weber *et al*. 2006).

## 4. The cyanobacterial pedigree of chloroplast two-component systems

Eukaryotes acquired two-component systems from the bacterial ancestors of chloroplasts and mitochondria through endosymbiotic, lateral gene transfer (Koretke *et al*. 2000). Sequence-similarity searches reveal homologues of the chloroplast two-component systems in extant cyanobacterial lineages, demonstrating the ancient origin of these systems from the cyanobacterial ancestor of chloroplasts. In *Synechocystis* sp. PCC 6803, histidine kinases 33 and 2 (hik33 and hik2) are homologues of ycf26 and CSK, respectively (Ashby & Houmard 2006; Puthiyaveetil *et al*. 2008). hik33 is also known as ‘drug sensory protein A’ (dspA; Hsiao *et al*. 2004; Morrison *et al*. 2005) in *Synechocystis* sp. PCC 6803 and as NblS in *Synechococcus elongatus* PCC 7942. Homologues of the chloroplast response regulators ycf27 and ycf29 are the response regulators 26 and 1 (rre26 and rre1) in *Synechocystis* sp. PCC 6803 (Ashby & Houmard 2006; Duplessis *et al*. 2007; Puthiyaveetil *et al*. 2008). TCP34, the nuclear-encoded response regulator-like protein of higher plant chloroplasts, does not appear to have cyanobacterial counterparts. The phylogenetic signature of this protein seems to have been lost during its evolution. TCP34 might represent a eukaryotic innovation in which the receiver domain of a response regulator is fused to a TPR, the latter motif being usually found in eukaryotes (Weber *et al*. 2006).

**Figure 5:**
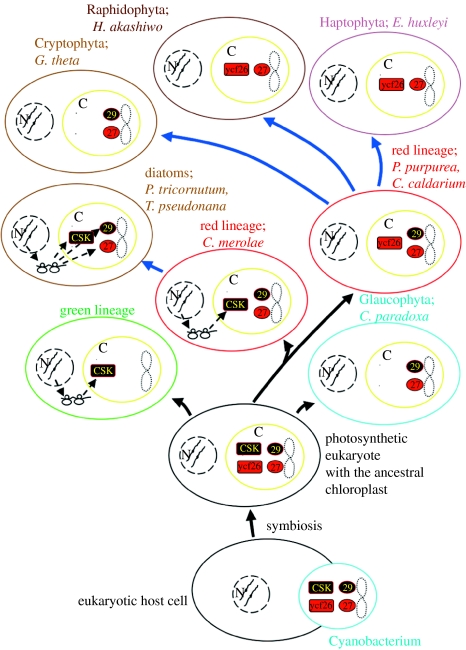
Lineage-specific distribution of the chloroplast two-component systems. Cyanobacteria (cyan) and chloroplasts (yellow) are represented as ovals. Names of lineages and their representative cells are coloured to correspond with their dominant pigments. Chloroplasts are additionally marked ‘C’ and the nucleus is represented by dashed circles and marked ‘N’. When genes encoding either or both component of the two-component systems are moved to nucleus, their synthesis in cytoplasm and import back to chloroplasts is indicated by ribosomes and dashed arrows. Lineages leading to diatoms, cryptophytes, raphidophytes and haptophytes have involved secondary endosymbiosis with a red algal symbiont and are represented by thick blue arrows from the red lineage. The numbers 27 and 29 denote the response regulators ycf27 and ycf29, respectively.

The presence of cyanobacterial homologues strengthens the genealogy of the chloroplast two-component systems, but can this information shed light on their structure and function? It is well known that the two-component systems usually work in cognate pairs to govern a functional response, even though cross-talk can occur in certain cases. In *Synechocystis* sp. PCC 6803, various functional and mutagenesis studies, together with high-throughput two-hybrid screening, have identified cognate pairs or interaction partners in different two-component systems (Murata & Dmitry 2006; Sato *et al*. 2007; Kappell 2008). At least two cognate pairs are important from a plastid perspective. These are the cognate pairs formed between hik33 and rre26, and between hik2 and rre1 (Paithoonrangsarid *et al*. 2004; Murata & Dmitry 2006; Sato *et al*. 2007; Kappell 2008). ycf26 and ycf27 form one cognate pair, while CSK and ycf29 together form the other pair. Although some chloroplasts still keep these ancestral combinations between sensors and response regulators, most chloroplasts have deviated from this pattern, for reasons that we do not yet fully understand. These deviations have resulted in a striking pattern of lineage-specific retention or loss of the chloroplast two-component systems (figure 5).

## 5. *Bricolage*. Lineage-specific retention and loss of chloroplast two-component systems

In photosynthetic eukaryotes, the chloroplast two-component systems as we understand them today consist of two sensors, two response regulators and a response regulator-like protein. The distribution of these proteins seems to follow a lineage-specific pattern (figure 5). In the lineage of rhodophytes leading to *P. purpurea*, *Cyanidium caldarium* and *G. tenuistipitata*, the chloroplast two-component systems consist of the ycf26 sensor and two response regulators, ycf27 and ycf29, all chloroplast-encoded (figure 5). In the raphidophyte *H. akashiwo* and the haptophyte *Emiliania huxleyi*, the same combination is seen except that the ycf29 response regulator is missing (figure 5). In the ancient red alga *C. merolae*, the response regulators are ycf27 and ycf29 proteins, both encoded in the chloroplast, while the sensor ycf26 is lost and presumably replaced by the nuclear-encoded CSK (figure 5). The glaucophyte *Cyanophora paradoxa* and the cryptophyte *Guillardia theta* resemble *Cyanidioschyzon* in their contingent of the chloroplast two-component systems (figure 5), but a nuclear-encoded CSK has yet to be demonstrated in their chloroplasts. Chloroplasts of bacillariophytes such as *P. tricornutum* and *T. pseudonana* do not encode any two-component system; nevertheless, they seem to contain the chloroplast two-component systems as products of nuclear genes, since homologues of *CSK*, *ycf27* and *ycf29* genes can be identified in their nuclear genomes (tables 1 and 2).

Chloroplasts in the green lineage (figure 5), with the single exception of *C. atmosphyticus*, seem not to encode the two-component systems. Homologues of CSK are readily identifiable in the sequenced nuclear genomes of higher plants, in the moss *P. patens* and in the prasinophycean alga, *O. tauri* (Puthiyaveetil *et al*. 2008). By contrast, chloroplast response regulators in green algae and land plants, again with the exception of *C. atmosphyticus*, remain unidentified. Whether the nuclear-encoded TCP34 protein can be counted as a genuine chloroplast response regulator remains to be seen.

Distribution of the chloroplast two-component systems appears to be a phylogenetic patchwork (figure 5). Nevertheless, a pattern emerges with regard to the location of genes encoding the chloroplast two-component systems. These genes are shown to move from chloroplast to nuclear genomes as we proceed from non-greens to greens (figure 5). Sequencing of more chloroplast and nuclear genomes may uncover yet further combinations of sensors and response regulators in chloroplasts. Genome sequencing will also answer questions such as the following: are there nuclear-encoded ycf26 homologues in algae and plants? Will a CSK homologue be found encoded in a chloroplast?

The phylogenetic distribution of the chloroplast two-component systems (figure 5) poses other unanswered questions. For example, why are non-greens reluctant to give up their chloroplast two-component genes to the nucleus, while greens are only too willing to do so? The answer to this question may lie in subtle differences between non-greens and greens in the structure and function of the chloroplast two-component systems.

## 6. The missing link between photosynthesis and gene expression

**Figure 6:**
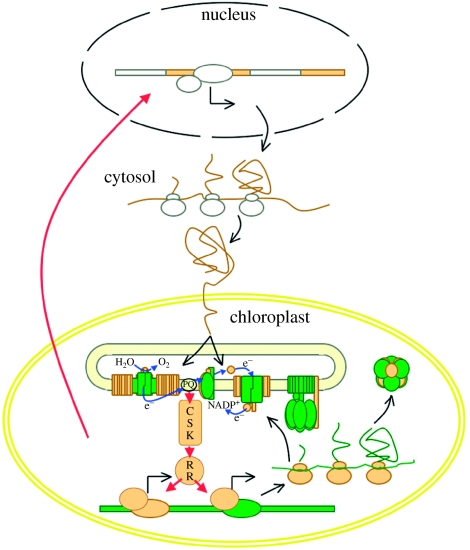
A model explaining the operation of a CSK-based two-component system in chloroplasts. CSK selectively switches on and off chloroplast genes in response to perturbations in the photosynthetic electron transport chain (depicted as electron transport from H_2_O to NADP^+^). CSK acts as a redox sensor and reports on electron flow. A response regulator protein (RR), the identity of which is yet to be revealed in green algae and plants, mediates CSK's control over gene expression. Chloroplast genes under CSK control are those which encode core components (shown in green) of the electron transfer complexes. The nuclear-encoded peripheral components are shown in yellow. The red arrow from the chloroplast to the nucleus represents plastid-to-nuclear signalling.

If two-component systems have survived in chloroplasts, the obvious question that comes to mind is ‘what do they do?’ Available data point to their indispensable role in linking photosynthetic activity to the expression of genes that encode for the photosynthetic machinery. The chloroplast two-component systems fulfil this vital role by acting as redox sensors and redox response regulators (Allen 1993*a*,*b*) inside the chloroplast (figure 6). CSKs, acting as redox sensors, monitor the flux of electron transport in the thylakoid membrane. The flux of electron transport lies at the heart of photosynthetic activity. Sensor kinases pass this information on to response regulators, which, when required, selectively turn photosystem genes on and off (figure 6). This feedback regulation brings about redox homeostasis by ensuring that photosynthetic activity in chloroplasts is rapidly and unconditionally optimized to new environmental conditions that have changed unpredictably. This proposition on the action of the chloroplast two-component systems is in agreement with their known functional properties.

Little is known concerning the action of the ycf26 sensor in non-greens. This is partly because knockout mutant lines are not available in these algae (Duplessis *et al*. 2007). However, if the perceived redox-sensing sequence features of this kinase are correct, then its stromally positioned PAS domain will act as a redox sensor domain, on its own or with the help of redox-responsive prosthetic groups (figure 2) such as flavins. As if to compensate for the lack of information on the action of ycf26 in non-greens, the role of hik33 in cyanobacteria is known in more detail. In cyanobacteria, hik33 is known to regulate photosynthetic gene expression in response to nutrient stress and high light intensities (Hsiao *et al*. 2004). hik33 is also known in cyanobacteria as a cold sensor (Mikami *et al*. 2003*b*), as a sensor of osmotic stress (Mikami *et al*. 2002; Paithoonrangsarid *et al*. 2004; Shoumskaya *et al*. 2005) and as a sensor involved in the expression of oxidative stress-inducing genes (Kanesaki *et al*. 2007). This multifunctional role could easily qualify hik33 as a global regulator in cyanobacteria. With its lumenal loop and PAS domain acting as two separate sensor domains, hik33 will be able to combine or integrate multiple signals and act as a multi-sensor (Morrison *et al*. 2005). Environmental factors such as low temperature, nutrient stress and osmotic stress may slow down photosynthetic electron transport, generating redox signals that will themselves feed into the regulatory loop of hik33. Additionally, the redox-sensing functional role of ycf26 is underscored by the observation that this sensor is seen in its minimal form, with only the PAS sensor domain and the kinase domain being present, in the raphidophycean alga *H. akashiwo* and in the cryptophycean alga *R. salina*.

For CSK, T-DNA insertion mutants are available in *Arabidopsis*, and their phenotype supports a role for CSK in coupling photosynthesis with chloroplast gene expression (Puthiyaveetil *et al*. 2008). This functional role of CSK was unravelled by the use of wavelengths of light preferentially absorbed by photosystem I (PS I) and photosystem II (PS II; Puthiyaveetil & Allen 2008*a*; Puthiyaveetil *et al*. 2008). Changes between lights of wavelength selectively absorbed by each photosystem affect the redox state of inter-photosystem electron carriers, including plastoquinone (PQ). The redox state of the PQ pool is a well-known regulatory signal affecting photosynthetic gene expression (Pfannschmidt *et al*. 1999*a*,*b*; Tullberg *et al*. 2000; Fey *et al*. 2005; Puthiyaveetil & Allen 2008*b*). Wavelength-dependent changes in chloroplast gene transcription are disrupted in CSK mutants, so CSK is proposed to act as a redox sensor that couples PQ redox state to gene expression in chloroplasts. In *C. merolae*, it has been shown that the mRNA of the *CSK* gene transiently accumulates after a switch to high light intensities (Minoda *et al*. 2005). CSK, like ycf26, shows redox-sensing sequence features, as it possesses a GAF domain at the N-terminus. GAF domains are known to sense redox signals (Kumar *et al*. 2007; Vuillet *et al*. 2007) and are structurally related to PAS domains (Ho *et al*. 2000). The cyanobacterial homologue of CSK, hik2, suggests functional properties that strengthen the redox-sensing functional role of CSK in chloroplasts. hik2 shows functional overlap with hik33 in sensing osmotic stress and low temperature (Mikami *et al*. 2003*b*). It has been further proposed that hik2 and hik33 are involved in the tolerance of PS II to environmental stress (Mikami *et al*. 2003*a*). Recent high-throughput yeast two-hybrid analysis has shown hik2 to interact with rppA (Sato *et al*. 2007), a redox response regulator in cyanobacteria (Li & Sherman 2000). This analysis also finds that hik2 interacts with the phycobilisome linker protein, apcE (Sato *et al*. 2007). These results are strongly indicative of a redox-sensing role for hik2 in cyanobacteria and, by extension, for CSK in chloroplasts.

The ycf27 response regulator in *C. merolae* binds upstream regions of the *psbD/C* operon and the *psbA* gene and is implicated in high-light acclimation (Minoda & Tanaka 2005). The cyanobacterial homologue of ycf27, rre26, is involved in the synthesis of factors responsible for coupling phycobilisomes to photosystems (Ashby & Mullineaux 1999). rre26 has recently been shown to bind the promoter region of the high-light-inducible hliB gene in *Synechocystis* PCC 6803 (Kappell & van Waasbergen 2007). Moreover, in cyanobacteria, the diverse physiological responses governed by the multi-sensor hik33 are mediated through the action of the response regulators rre26 and rre31 (Paithoonrangsarid *et al*. 2004; Murata & Dmitry 2006; Kappell 2008).

The ycf29 response regulator binds genes encoding the components of light-harvesting phycobilisome proteins such as cpcA and apcE (Minoda & Tanaka 2005). Transcriptional activation of these genes by ycf29 is believed to be part of an acclimatory response to decreased light intensity (Minoda & Tanaka 2005). In cyanobacteria, the action of hik2 is thought to be mediated through rre1, the ycf29 homologue (Paithoonrangsarid *et al*. 2004). TCP34, the response regulator-like protein of land plants, has been shown to bind *psaA*, *rbcL*, *psbC* and *psbD* gene core promoters in spinach chloroplasts (Weber *et al*. 2006).

It has been suggested that the distribution of ycf27 genes is somehow correlated with the retention or loss of phycobilisomes in various algal groups (Ashby *et al*. 2002). The loss of ycf27 genes from plastids has been linked with the loss of phycobilisomes. But the demonstration of ycf27 genes in the non-phycobilisome-containing genera (table 2) *H. akashiwo*, *G. theta*, *E. huxleyi* and *C. atmosphyticus* proves that no such link with phycobilisomes is required for the retention of ycf27 genes (Duplessis *et al*. 2007).

Our own analysis shows that the loss of the ycf29 response regulator gene is not linked with the loss of phycobilisomes as ycf29 genes are present in non-phycobilisome-containing species such as the diatom *T. pseudonana* and the cryptophyte *G. theta* (table 2). Nonetheless, there may exist some functional link between ycf29 and phycobilisomes. For instance, the ycf29 response regulator is involved in transcriptional activation of phycobiliproteins genes as part of an acclimatory response to low light intensity in *C. merolae* (Minoda & Tanaka 2005). The demonstration that ycf27 and ycf29 genes are retained in non-phycobilisome-containing algae is a clear indicator that these two response regulators have other target genes in chloroplasts.

## 7. Why are there genes in the cytoplasm?

The two-component systems connect photosynthesis to gene expression, but how exactly does this connection bring about homeostasis in chloroplast function? The answer lies in the chloroplast's resulting ability to control biogenesis of the electron transfer complexes participating in the light reactions of photosynthesis (Allen 2003). These electron transfer complexes are multi-subunit pigment–protein assemblages with their core protein components being encoded by the chloroplast genome and the peripheral protein components encoded by the nuclear genome. The biogenesis of these electron transfer complexes involves a hierarchical assembly in which the chloroplast-encoded core components are made and inserted into the thylakoid membrane first, followed by the assembly of the nuclear-encoded peripheral components (Choquet & Vallon 2000).

Some general principles have been recognized in the highly concerted assembly processes of electron transfer complexes in chloroplasts (Choquet & Vallon 2000). Nuclear-encoded subunits are found to be less essential to the stability of the complexes than the chloroplast-encoded core subunits. Among chloroplast-encoded subunits, there exists a sequential and ordered assembly that arises from a hierarchical organization in the expression of these subunits. In regulatory terms, this means that the translation of certain subunits is controlled by the state of assembly of the complex. To be precise, the rate of translation of some subunits depends on other subunits being present in the membrane. The former subunit is called a CES (control by epistasy of synthesis) protein and the latter is a dominant assembly factor (Choquet & Vallon 2000). The dominant assembly factors are less stable in the complexes and, in the absence of their assembly partners, are rapidly degraded by proteases. CES proteins on the contrary are more stable, but their synthesis is reduced in the absence of dominant assembly factors, by an autoregulatory translational loop. In *Chlamydomonas*, mutant studies have revealed that, in each complex of the thylakoid membrane, at least one subunit is under epistatic control of synthesis and is thus a CES protein. Cytochrome *f* in cytochrome *b*_6_*f*, psaA protein in PS I, D1 and CP47 in PS II, *α* subunit in ATP synthase and the large subunit of Rubisco are CES proteins (Choquet & Vallon 2000).

Control by epistasy of synthesis and biogenesis of electron transfer complexes, in general, offer some important insights into the gene content of chloroplast genomes and the genetic control mechanism of photosynthetic genes. Autoregulation of translation of CES proteins becomes possible only when their genes are present in the same location as where the assembly takes place (Choquet & Vallon 2000). CES properties certainly explain why genes encoding CES proteins should be present in chloroplasts, but they do not explain why genes encoding dominant assembly factors should also be present in chloroplasts. The retention of these latter genes, however, has to do with the nature of the biogenesis process itself. A process in which the chloroplast-encoded core subunits act as ‘seeds’ or ‘nuclei’ on which the nuclear-encoded peripheral subunits assemble to form functional electron transfer complexes.

The pivotal role of chloroplast-encoded subunits in assembly is further highlighted by the observation that some small subunits are encoded in the chloroplast genome and integral to assembly without forming part of a mature, functional complex (Boudreau *et al*. 1997). The pre-eminence of chloroplast-encoded subunits in the assembly process means that, if chloroplasts can rapidly control the availability of core subunits, they can ‘make’ or ‘break’ whole-electron transfer complexes. The precisely timed delivery of assembly-competent, chloroplast-encoded core subunits is essential not only for the assembly process itself, but also for the prompt binding of chlorophylls and other cofactors that are synthesized in the chloroplast. The deleterious effects of free chlorophylls and their biosynthetic intermediates are well known.

Control over the availability of chloroplast-encoded core subunits can be achieved by means of both transcriptional and translational regulation, although the former has been considered the principal mode of gene regulation. Since chloroplast-encoded core subunits consist of both dominant assembly factors and CES proteins, it is interesting to ask whether both classes of genes are under transcriptional control. If both categories of genes are under transcriptional control, will they differ in their levels of response? For example, will one class of genes respond faster than the other? The polycistronic nature of many chloroplast mRNAs suggests that both dominant assembly factor and CES protein genes could be equal targets of transcriptional regulation.

If rapid regulation of chloroplast-encoded genes is what tips the equilibrium of complex formation, how will chloroplasts know which chloroplast genes should be turned on and which turned off? The redox state of photosynthetic electron carriers is a source of information for deciding the composition of thylakoid membranes. However, this decision will require a continuous dialogue between electron carriers and chloroplast genes encoding core subunits. A minimal signal transduction chain in the form of a two-component system makes this dialogue possible. The chimeric nature of the electron transfer complexes further requires that the dialogue within the chloroplast be overheard by the nucleus, so that the availability of the nuclear-encoded peripheral subunits is also, eventually, brought under control. Plastid-to-nuclear signalling pathways (Gray *et al*. 2003; Fey *et al*. 2005; Lopez-Juez & Pyke 2005; Koussevitzky *et al*. 2007; Moulin *et al*. 2008) must have originated to achieve the balanced coordination that is required for the assembly of functional electron transfer complexes.

It has been suggested that the direct and rapid redox regulation of genes encoding core subunits of the electron transfer complexes by the process of redox chemistry is the reason why chloroplasts and mitochondria retain functional genomes (Allen 1993*a*; Allen 2003). As is evident from the biogenesis of electron transfer complexes, rapid regulation of chloroplast-encoded core subunits is required for the ordered assembly of electron transfer complexes. The property of the co-location of genes and gene products for redox regulation (CoRR) appears to be inseparably integrated into the assembly and biogenesis of electron transfer chains. This means that the gene content of chloroplast genomes is explained only when the CoRR hypothesis (Allen 2003; box 1) is viewed in the context of the biogenesis and assembly processes of electron transfer complexes. A synthesis of CoRR (Allen *et al*. 2005; box 1) and biogenetic principles might suggest that species-specific differences in assembly processes result in species-specific differences in the gene content of chloroplasts. An example of such difference is the observation that photosystem genes such as *psaD*, *psaE* and *psaF* are always chloroplast-encoded in red algae, but they are nuclear-encoded in green algae and plants.

## 8. Amplification, gain control and an autonomous clock

The emerging properties of the chloroplast two-component systems and their cyanobacterial homologues demonstrate their role in linking photosynthesis with gene expression. How does this general function account for the uneven distribution of their genes between chloroplast and nuclear genomes? One hypothesis is that the retention of two-component genes in chloroplasts confers a selective advantage to the organism bearing them, since they may then be able to mount a rapid and elaborate adaptive response to serious environmental assaults such as high light intensities (Allen 2003). This amplified response is thought to be mediated through a positive feedback loop in the two-component regulatory system, since its components are encoded in the operon that they control (Allen 1995; Allen & Nilsson 1997). Such a positive feedback loop has been shown to promote a transcriptional surge that jump-starts virulence in *Salmonella typhimurium* (Shin *et al*. 2006). Amplification of signals within chloroplasts may also explain the occurrence of chloroplast-encoded response regulator genes in more than one copy in some non-green algae. This proposal (Allen 1995) is also in agreement with the demonstrated high-light acclimatory roles of the chloroplast two-component systems and their cyanobacterial homologues (Hsiao *et al*. 2004; Kappell & van Waasbergen 2007).

Being part of the operon that it controls is also a characteristic feature of the transcriptional feedback loop in circadian oscillators. We propose that the circadian analogy can be applied to chloroplasts, and that the chloroplast-encoded two-component systems, acting as endogenous oscillators, generate a rhythmic pattern of chloroplast mRNA accumulation. A diurnal rhythm has been noted in photosynthetic electron transport (Okada & Horie 1979). The chloroplast two-component systems, as discussed earlier, connect the activity of photosynthetic electron transport chain to gene expression in chloroplasts. Thus, it is likely that an autoregulatory loop in the transcription of chloroplast-encoded two-component systems, when connected to the rhythmic activity of photosynthetic electron transport, will generate a rhythmic transcript accumulation pattern for chloroplast genes.

An endogenous rhythm of chloroplast transcript accumulation has been demonstrated (Doran & Cattolico 1997) and is thought to be driven by the activity of the photosynthetic electron transport chain. If our proposal is correct, chloroplasts in the green lineage may have lost this transcriptional loop as genes encoding the chloroplast two-component systems have moved to the nuclear genome. The probable existence of a post-translational feedback loop in the nuclear-encoded chloroplast two-component systems may still ensure an endogenous rhythm of transcript accumulation in chloroplasts, as has been observed in *Arabidopsis* (Puthiyaveetil & Allen 2008*b*). Additionally, the observation that, in *CSK* null mutants, rhythmicity of *psaA* transcript accumulation has been greatly attenuated (Puthiyaveetil *et al*. 2008) supports the role of a nuclear-encoded chloroplast two-component system in rhythmic transcriptional activity of chloroplast genes.

The endogenous rhythm in chloroplasts is distinct from the circadian rhythm in that the former is aperiodic in oscillation. The aperiodicity in the chloroplast's endogenous rhythm was demonstrated in algae, where the endogenous rhythm of transcript accumulation shows a temporally gated response to changes in photoperiod (Doran & Cattolico 1997). Doran & Cattolico (1997) showed that the ability of the algae to increase the amplitude of the transcriptional response in response to the changes in photoperiod was limited to the first 2 hours of the dark period. Likewise, in the red alga *C. merolae*, a similar chloroplast transcriptional response was noted in response to shifts from dark to light conditions (Minoda *et al*. 2005). Minoda *et al*. (2005) found that the transcription of photosystem genes and other chloroplast genes encoding core subunits of electron transfer complexes peaked 1 hour after dark to light switch, and then decreased for 6 hours. One can easily envisage how an endogenous oscillator made of the chloroplast two-component systems can generate such aperiodic transcriptional rhythms in chloroplasts.

There may be clear advantages for the endogenous rhythm of transcript accumulation in chloroplasts not being circadian. For example, chloroplasts are faced with aperiodic fluctuations in incident light quantity and quality, such as those caused by cloud cover or by transient shading by the leaf canopy. For aquatic algae, changes in position in the water column also constitute a major source of variability in light quantity and quality. In order to improve photosynthetic efficiency and to avoid free radical generation by inadvertent electron transfer reactions under such conditions, chloroplasts can rapidly tune the expression of genes encoding core subunits of the electron transfer complexes with the help of an aperiodic clock. As seen in the earlier section, the chloroplast's ability rapidly to regulate the expression of chloroplast-encoded core subunits gives them the upper hand in the assembly of electron transfer complexes, and hence in altering thylakoid composition. The chloroplast's endogenous rhythm in transcript accumulation is thus driven by the availability of light and can be best described as a dial, or light–dark, cycle.

## 9. Phase differences as the dialogue between nuclear and cytoplasmic genetic systems

It therefore appears that, as well as providing the redox-signalling device that connects photosynthesis with gene expression, the two-component systems have endowed chloroplasts with an autonomous clock. A common evolutionary origin of biological redox sensors and biological chronometers can be envisaged in the context of light–dark cycles acting upon photosynthesis: an ancestral redox sensor of photosynthetic electron transport may have diverged to form both a redox-signalling system entrained by light and, eventually, a parallel, free-running system recording the passage of time (Allen 1998).

Synchronization of the chloroplast clock with the nuclear–cytosolic clock may represent an ideal steady state under constant environmental conditions. However, an essential feature of all living cells is homeostasis—their capacity to adjust to a changing external environment and so maintain a constant internal environment. We suggest that phase differences between two chronometers underlie signalling between the chloroplast and the nucleus. A photosynthesis- and redox-entrained chloroplast clock may depart in phase from a photoreceptor-entrained and nuclear–cytosolic clock. We suggest that the regulatory coupling between nuclear and chloroplast genetic systems illustrated in figure 6 may result from, and correct, the phase differences between their two clocks.

## 10. Conserved signalling throughout a major evolutionary transition

The two-component systems in chloroplasts persist from the ancestral cyanobacterial endosymbiont and thus epitomize a signalling system that could never have been put ‘on hold’ in evolution. Close coupling of photosynthesis with gene expression using a two-component system was vital before, during and after the transition of cyanobacterium to chloroplast. We now need to understand the precise nature of the redox signals sensed, the mechanism of signal perception by sensor kinases, the identity of their cognate response regulators and the interaction of these response regulators with their target genes. The two-component systems common to chloroplasts and cyanobacteria are keys to understanding the link between photosynthesis and gene expression, and may also illuminate the consequences of this link for eukaryotic cell evolution (Allen *et al*. 2008).

## Figures and Tables

**Table 1:** Distribution of chloroplast sensor kinases in photosynthetic eukaryotes. (A tick (√) indicates the presence and a cross (×) indicates the absence of ycf26 or CSK. A dash (—) indicates that the complete genome sequence for that taxon is not available, so the presence or absence of ycf26 or CSK is unknown. An asterisk (^*^) denotes that the occurrence is based on an earlier report (Duplessis *et al*. 2007). The taxonomic group ‘Viridiplantae’ means ‘green plants’, and includes green algae, lower plants and higher plants.)

taxonomic group/organism	ycf26	CSK
glaucophytes		
*Cyanophora paradoxa*	×	—
rhodophytes		
*Porphyra purpurea*	√^*^	—
*Porphyra yezoensis*	√^*^	—
*Porphyridium aerugineum*	—	—
*Cyanidioschyzon merolae*	×	√
*Cyanidium caldarium*	√^*^	—
*Galdieria sulphuraria*	—	—
*Gracilaria tenuistipitata*	√^*^	—
*Rhodella violacea*	—	—
haptophytes		
*Emiliania huxleyi*	√^*^	×
raphidophytes		
*Heterosigma akashiwo*	√^*^	—
cryptophytes		
*Guillardia theta*	×	—
*Rhodomonas salina*	√	—
bacillariophytes		
*Phaeodactylum tricornutum*	×	√
*Thalassiosira pseudonana*	×	√
viridiplantae		
*Ostreococcus tauri*	×	√
*Ostreococcus lucimarinus*	×	√
*Chlamydomonas reinhardii*	×	×
*Chlorokybus atmosphyticus*	×	—
*Physcomitrella patens*	×	√
*Pinus taeda*	×	√
*Oryza sativa*	×	√
*Arabidopsis thaliana*	×	√

**Table 2:** Distribution of chloroplast response regulators and response regulator-like proteins in photosynthetic eukaryotes. (A tick (√) indicates the presence and a cross (×) indicates the absence of ycf27/ycf29/TCP34. A dash (—) indicates that the complete genome sequence for that taxon is not available, so the presence or absence of ycf27/ycf29/TCP34 is unknown. An asterisk (^*^) denotes that the occurrence is based on earlier reports (Weber *et al*. 2006; Duplessis *et al*. 2007). Chloroplast response regulators are mostly chloroplast gene products, but if the chloroplast response regulators exist as nuclear gene products, the nuclear location of the corresponding gene is indicated by a superscript N (^N^).)

taxonomic group/organism	ycf27	ycf29	TCP34
glaucophytes			
*Cyanophora paradoxa*	√^*^	√	—
rhodophytes			
*Porphyra purpurea*	√^*^	√	—
*Porphyra yezoensis*	√^*^	√	—
*Porphyridium aerugineum*	√^*^	—	—
*Cyanidioschyzon merolae*	√^*^	√	×
*Cyanidium caldarium*	√^*^	√	—
*Galdieria sulphuraria*	√	—	—
*Gracilaria tenuistipitata*	√^*^	√	—
*Rhodella violacea*	√^*^	—	—
haptophytes			
*Emiliania huxleyi*	√^*^	√^N^	×
raphidophytes			
*Heterosigma akashiwo*	√^*^	×	—
cryptophytes			
*Guillardia theta*	√^*^	√	—
*Rhodomonas salina*	√	√	—
bacillariophytes			
*Phaeodactylum tricornutum*	√^N^	√^N^	×
*Thalassiosira pseudonana*	√^N^	√^N^	×
viridiplantae			
*Ostreococcus tauri*	×	×	√^N^
*Ostreococcus lucimarinus*	×	×	√^N^
*Chlamydomonas reinhardii*	×	×	×
*Chlorokybus atmosphyticus*	√^*^	×	—
*Physcomitrella patens*	×	×	√^N^
*Pinus taeda*	—	—	—
*Oryza sativa*	×	×	√^*N^
*Arabidopsis thaliana*	×	×	√^*N^

## Box 1

The CoRR (co-location for redox regulation; Allen 2003) hypothesis states that genes have been retained in bioenergetic organelles to provide the direct and unconditional regulation of their expression by the redox state of their gene products, the core proteins of energy transduction in photosynthesis and respiration. Six out of the ten axioms and predictions of CoRR (Allen *et al*. 2005) apply directly to our present knowledge of the chloroplast two-component systems.

- *The principle of selective value of redox control.* For each gene under direct redox control, it is selectively advantageous for that gene to be retained and expressed only within the organelle.
- *The principle of unselective gene transfer and protein import.* Gene transfer from the organelle to the nucleus of the host cell is not selective for particular genes and there is no barrier to the successful import into the organelles of any precursor protein.
- *The principle of selective value for nuclear location of genes.* It is selectively advantageous to relocate those organellar genes that do not encode core subunits of electron transfer complexes, and for which expression need not be redox-regulated, to the nucleus of the host cell.
- *The principle of primary involvement in energy transduction.* Those genes for which direct redox control is vital, and for which gene products form the core subunits of the electron transfer complexes and participate in primary electron transfer reactions, are always contained in the organelles.
- *The principle of secondary involvement in energy transduction*. Genes for which products constitute the organelle genetic system, or for which products are associated with secondary events in energy transduction, may be retained in organelles in one group of organisms, but not in another.
- *The principle of nuclear encoding of redox-signalling components*. The redox signalling components, upon which the co-location of genes and gene products is based, are themselves not involved in primary electron transfer, so their genes have been relocated to the nucleus.
